# Supplementary material for: Micafungin-Induced Cell Wall Damage Stimulates Morphological Changes Consistent with Microcycle Conidiation in Aspergillus nidulans
Source: J Fungi (Basel). 2021 Jun 29;7(7):525. doi: 10.3390/jof7070525 (PMC8306900; doi:10.3390/jof7070525)
Supplement: Supplementary file 1 [file jof-07-00525-s001.zip › Supplemental Figures_V3/Supplemental Figure S1 Shared DE Genes.pdf]

**A**

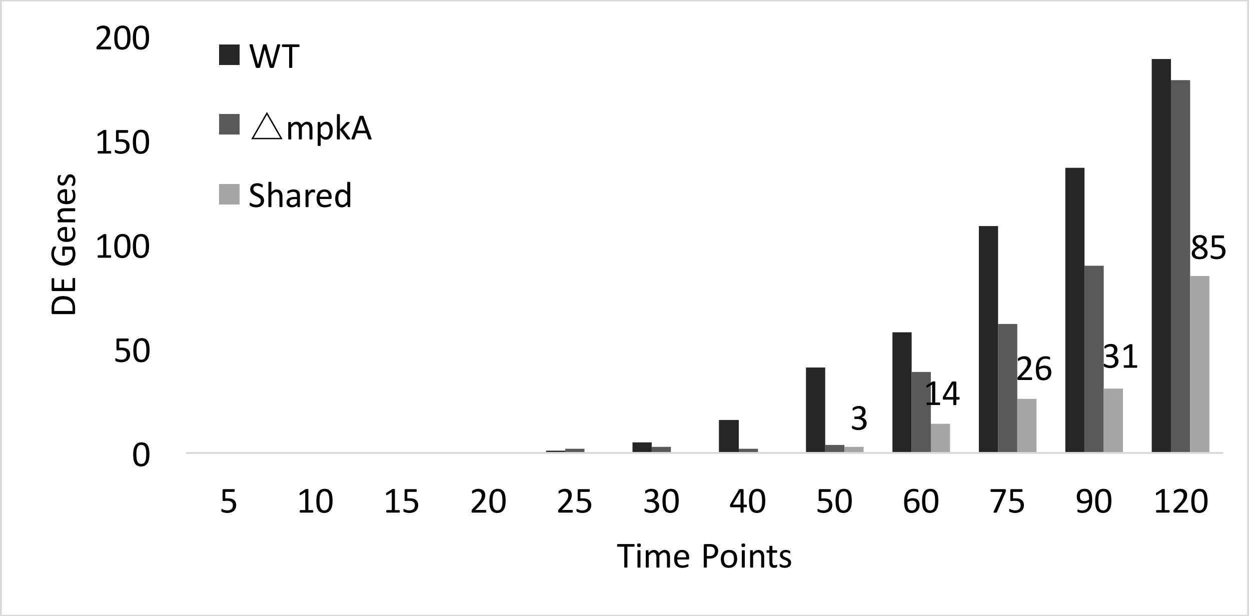


**B**

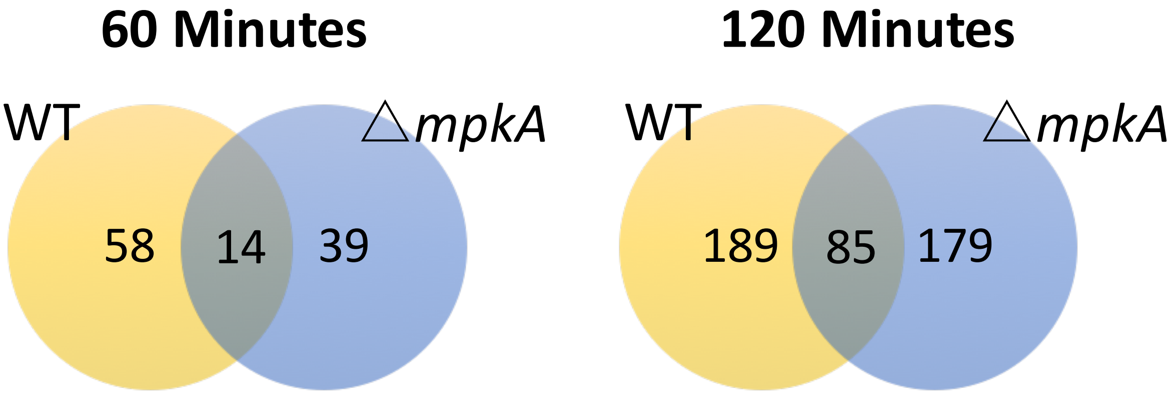


**Supplemental Figure S1.** **A** Up-regulated DE genes that were shared by both wildtype and Δ*mpka* through all the time points. The first shared gene occurs at 50 minutes. **B** Time point 60-minutes there are 14 shared DE genes and at time point 120 minutes there are 85 shared genes
