# Supplementary material for: Micafungin-Induced Cell Wall Damage Stimulates Morphological Changes Consistent with Microcycle Conidiation in Aspergillus nidulans
Source: J Fungi (Basel). 2021 Jun 29;7(7):525. doi: 10.3390/jof7070525 (PMC8306900; doi:10.3390/jof7070525)
Supplement: Supplementary file 1 [file jof-07-00525-s001.zip › Supplemental Figures_V3/Supplemental Figure S2 BrlA42 Mutant.pdf]

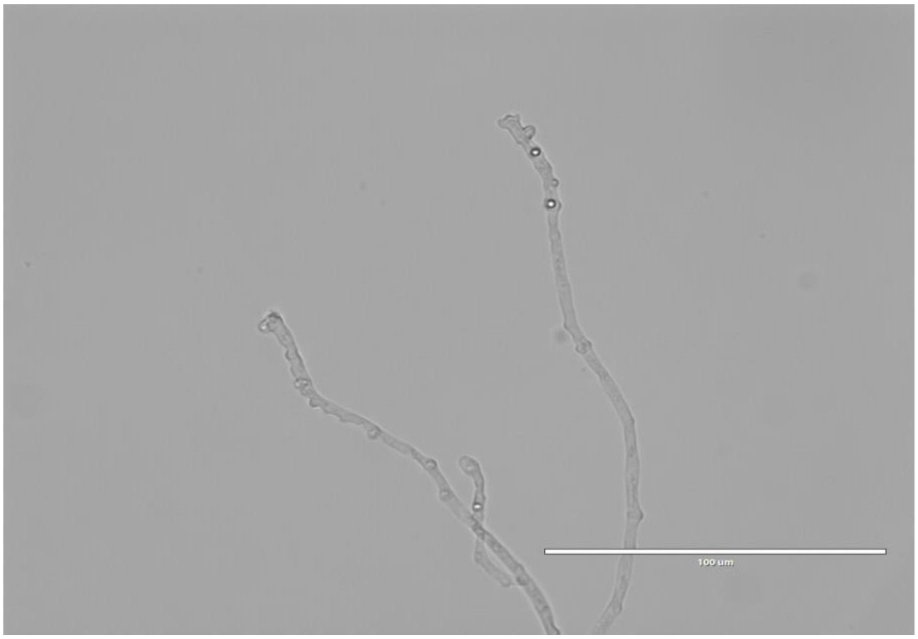

<u>Supplemental Figure S2</u>. *brlA42* mutant eleven hours post 0.1ng/ml micafungin exposure. 40X magnification. Scale bar is 100µm. Pictures taken on EVOS FL microscope.
